# Supplementary material for: A reduced state of being: The role of culture in illness perceptions of young adults diagnosed with depressive disorders in Singapore
Source: PLoS One. 2021 Jun 9;16(6):e0252913. doi: 10.1371/journal.pone.0252913 (PMC8189483; doi:10.1371/journal.pone.0252913)
Supplement: S1 File — (DOCX) [file pone.0252913.s001.docx]

**S1 File. Interview Schedule**

A. General information

1. Can you tell me a bit about yourself so I can know you better?

B. Illness perception

1. Could you tell me a bit about depression?

a. *Do you check online to find out more about depression?*

*b. (If Yes, and P mentions social media platforms) How did social media* influence your understanding of the illness?*

2. (General) In your opinion, why do people become depressed?

3. (Specific to own experience) In your opinion, and it is ok if it is different from what your doctor believes about the condition- what do you personally believe caused your illness?

4. How have you coped with depression? Could you elaborate on that?

5. How long do you think depression will last? What makes you think this way?

6. How has depression affected your life?

7. How do you think depression is curable? Could you elaborate further?

D. Culture and ethnic background

1. What does your family think about depression or mental illness in general?

2. What do other people belonging to the same cultural background as you think about depression or mental illness in general?

3. Has your family and/or culture affected the way you think about the illness?

4. Have you gone for any other forms of treatment for your illness? What made you go there?

*Note: Questions 1a and 1b were added into the interview schedule after the theme of social media emerged in some narratives (not reported in this manuscript).*
