# Supplementary material for: A reduced state of being: The role of culture in illness perceptions of young adults diagnosed with depressive disorders in Singapore
Source: PLoS One. 2021 Jun 9;16(6):e0252913. doi: 10.1371/journal.pone.0252913 (PMC8189483; doi:10.1371/journal.pone.0252913)
Supplement: Attachment — Submitted filename: Response to Reviewers.docx [file pone.0252913.s002.docx]

18 November 2020

RE: Responses to reviewers

Dear Reviewers,

We thank you for taking the time to review our manuscript. We have learnt a lot from the peer review process and have incorporated your comments in our next draft. Please find our point-by-point responses to the comments in the boxed text area below:

**Reviewer #1**: This paper presents an original qualitative research on illness perceptions in young adults diagnosed with depressive disorders based on an Asian context (Singapour). Overall, the topic is highly relevant for psychology and the qualitative perspective s most appropriate to explore narratives to understand the lived experience of people with depressive disorders. The paper is well-structured and the English is correct.

Despite these qualities, the current version of the paper calls for substantial changes at epistemological, theoretical and methodological levels in order to be potentially ready for publication in PLOS ONE, especially given the solid and high quality of this journal. Here below my suggestions to improve the quality of the present version of the manuscript:

1) Introduction: The Leventhal model is presented in the introductory part as a core theoretical perspective mobilized by the authors, as they position themselves critically towards such model. However, the reader wonders why, given the broad body of qualitative research on depression, the introduction is narrowed down to research on this model? What about other qualitative approaches to depression, that also take into account narratives on illness (ex. phenomenology and IPA, narrative, discursive...).

Thank you for this. We agree with the reviewer that more could be elaborated on this point. There are important reasons for using Leventhal’s model. Firstly, the Leventhal model is one of the most widely used model due to its good psychometric properties, to predict self-care behaviours and recovery in physical illnesses. Secondly, unlike other health belief models, this model comprises of emotional illness representations, which are directly relevant to mental illnesses. We have elaborated on this point in the subsequent draft.

We agree that there are many other qualitative approaches that are highly relevant to the study of narratives. For instance, IPA is very useful in providing rich accounts of the depression experience. However, as it had been previously discussed by Braun and Clarke (2020) and Chamberlain (2012), there is no one ideal or superior qualitative method. The choice of method may also be dependent on pragmatic factors as well- we are unfortunately limited in our access to resources and are facing time constraints. Finally, authors (and coders of this study) are experienced and trained in thematic analysis only. We wish to be able to use other qualitative approaches in the near future because of its relevance in the work that we do. We agree with the reviewer that this was insufficiently justified in the manuscript and have made substantial edits to it.

I very much appreciated that you mention the role of culture as it shapes the experience of illness - This is an inherent part of the socio-constructionist perspective namely. But scientific references are missing to better argue on the crucial role of culture (understood as a set of norms and values - giving rise to social narratives that contribute to determine how we experience the world and ourselves). The distinction between West and East remains rather superficial and broad. What seems specific to the context you chose for your research and why is it relevant for the hypothesis that you raise at the end of this section?

Thank you for this. As the reviewer had succinctly pointed out, the illness experience of an individual may be influenced by societal norms and values. We agree fully with the reviewer that this portion is underdeveloped in the present draft, and have since made changes in the subsequent draft.

Furthermore, while narratives seem the crucial focus on this paper, the reader cannot really grasp from the manuscript's content why the narrative focus is so important. As a qualitative researcher in psychology, I personally fully agree with you on the central role of narratives. But why are narratives so important? Different perspectives have been developed within psychology, namely that of structuring our sense of selves and our world (discursive, constructionist, narrative), but also that of making sense of our experience (for example, phenomenology). However, the choices underlying your theoretical approach seem absent /scarcely developed. Indeed, there are multiple approaches to narratives - and this theoretical foundation is currently missing from your paper. The problematisation at the end of the introduction falls short: why the hypothesis on the sense of self? Can you develop what you mean by this and argue with additional references to make your research/theoretical posture more explicit?

As quantitative researchers by training, we are largely unfamiliar with the theoretical concepts that are widely used in sociology. However, social constructivism, as we recognize, is highly relevant to our study. Thus, we understand and agree fully with the reviewer that the introduction can be improved further by laying theoretical grounds for the study and we hope the subsequent draft reads better. We thank the reviewer for these suggestions.

2) Methodology: To me, this section requires important changes. Under the 'procedure' paragraph, the population is already mentioned but then you come back to participants in the next paragraph entitled 'participants' - The reader can be a bit lost in this order. I would suggest to better justify the qualitative approach used in the 'procedure' paragraph to better explain the whole methodological procedure in a general way.
line 119: what do you mean by 'subconsciously'? not appropriate from my perspective - better explain your decision on the context of the interviews

Thank you for this. The main reason for carrying out the interviews away from the clinical setting was to prevent contextual priming at a subconscious level that may influence the way participants describe their illness experience clinically during the interviews. We have rephrased this explanation and restructured the paragraphs.

In the population paragraph, the average age needs to be better justified - (especially if 'young adults' is part of your title - why young adults rather than older adults? Also, there is no mention of the gender except for the number of male and female - but according to me, the 'gender' dimension needs to be at least considered and justified to increase methodological rigor - as in depression this seems to play an important role (ex. constructions of masculinity/feminity)

Major Depressive Disorder is the most prevalent mental illness among young adults aged 18 to 34 years old (Subramaniam et al., 2020), thus, these narratives describe how depression is typically experienced at the onset for this age group. Understanding how illness is experienced among young adults would be important for treatment and recovery that is also relevant for this age group.

Before the start of the study, researchers conducted a literature review of illness perception of severe mental illnesses. As reported in the introduction, the primary themes that emerged were related to the self, with no prominent mentions of gender. During the coding and analyses phase, the first author did suspect possible cultural influences on the role of gender. For instance, the theme “High societal expectations of success”, initially emerged from the narratives of male participants. This was detailed in her memo, indicating that instances like this could demonstrate cultural expectations of the need to succeed being more salient in men. However, during the course of coding, this theme was prominent in female participants as well. While we agree with the reviewer that gender does play an important role, it did not emerge during the literature review and analyses.

Also, on the interview description - there is no information on the interview guide nor how it was elaborated. This is an important aspect to better understand how the analysis were conducted afterwards, in the analytical phase.

Thank you for this, the interview guide has been attached as supporting information in the subsequent submission.

Regarding the 'analysis' section - practically no scientific references are used to justify your analysis technique. Yet, this methodological stage is particularly important. I would suggest reading Virginia Braun and Victoria Clarke who have extensively written on thematic content analysis. Line 144: Saturation? Better explain and use scientific reference (This notion is contested in qualitative research depending on the authors). Line 158: open and ground coding - what does this mean? Reference please

Thank you for this. We recognize that this is important but was mistakenly left out. We have uploaded the interview schedule in its final form. We have also improved on the analysis section to explain our sampling and analysis technique, including scientific references to support our justifications.

3) Results: The themes are very general - A 'good' theme definition is that which captures in its name the full condensed 'story' or 'message' that the given set of data portrays.
Moreover, please restructure with numbers or letters in hierarchical order the different themes and subthemes as it is difficult for the reader to follow. I regret that there is a lot of 'verbatim' that illustrate the names of your subthemes but little explanation or interpretation on what these themes and subthemes mean in relation to your hypothesis and research aim. At times, the text is written as if narratives 'reflected' a 'truth' or a 'reality' - Please keep in mind what status do narratives have in your theoretical perspective to better guide you on how to write about them.

Thank you for the suggestions. We have made additional changes to the name of the themes/subthemes to better represent the themes that emerged from the narratives.

I sometimes disagreed with the authors' explanation on certain quotes - to me, Line 200 for instance, would call for a separation between mind and body rather than depression as a different entity, from what I understand from the participants' verbatim

The verbatim quote may not have clearly captured the context. What we had understood from the participant during the interview was that “…the thing is in your mind. So *[the thing in]* the mind is taking over your body…” M11/31/F/M

Often, authors refer to results as 'they' (participants') as if all participants had positioned themselves in an homogeneous way regarding their experience. Were there any dissident narratives for example?

Thank you for this. Only the prominent themes were reported in the manuscript. The prominence of themes was not necessarily dependent on the majority although it was primarily so, and we would have stated if the themes or codes emerged only from a few. For instance, the narrative that depression is separate from or a part of the self, came from two different groups of participants, which we had merged to form a subtheme. We did encounter dissident narratives, for instance, narratives like, “*depression comes from the brain so I think to actually rewire something takes a long time M12/26/F*”, or *“depression is something that’s very, very repetitive C12/22/F*”, but we did not report as it was decided among coders that they were not prominent codes.

Line 286: while this seems a key theme (or subtheme?) it is very little developed/explained.
Line 315: spelling of 'yeah'

Line 352: This theme follows a hierarchical numeration while the rest of the themes do not.
In the end of the result section, a synthesis of results would be very useful.

Thanks for highlighting as these were mistakes. We have edited/removed them in the subsequent draft. The format of the manuscript has changed, and we hope it will read better.

Discussion: Coming back to the CSM model - why and how it was confirmed? Why do authors overlook the similarities of their results with the model (if it's an important focus of the paper)?

Thank you for this. The main objective of the study is to understand illness perceptions of depressive disorders, and it was not to disprove or confirm the CSM model. We had however, made few concluding statements as our study provided support for the conclusion that the CSM may be overly simplistic, although it is a useful guide for mental illnesses.

Line 428 'are under pressure' - this is a strong affirmation that is different from 'perceive themselves as being under strong pressure' for example. This study is qualitative so it takes the subjective constructions into account - not revealing an 'objective' reality.
Line 474: regarding the limitations, please consider that the aim of qualitative research is not generalization but on the contrary, considering variablity, contextualization, narratives, singularity, experience - therefore it is not a limitation from my view.

We agree with the reviewer and had removed this statement from limitations. We thank the reviewer for this important point.

Finally, how can your conclusions be extended beyond the Leventhal model and contribute to psychology more broadly? This could be a stronger case if your theoretical position/framework were better made explicit in the introduction for example.

The themes that emerged in this study were highly associated with the self, emphasizing the importance of self-concepts, such as self-agency, goals, sense of meaning and belonging, in the study of illness perceptions of severe mental illnesses. Additionally, our results evidently show that personal recovery requires the consideration of cultural beliefs that tie closely to illness beliefs among those surviving with depressive disorders in Singapore.

Thank you for taking the time to review this article. We hope the subsequent draft reads better.

**Reviewer #2**: Thank you for asking me to review this manuscript.
This qualitative inquiry aimed to explore the illness perceptions of Chinese, Malay, and Indian young adults diagnosed with depressive disorders by using one-to one face-to-face semi-structured interviews. They concluded that depression was typically experienced as a reduced state of being, and was thought of cognitively as an entity that may be a part of or separate from the self. Five themes were identified as: 1) meanings, 2) causes, 3) symptoms, 4) consequences, and 5) chronicity of depression.

Such an effort could be valuable for cultural diversity and future implication. The following issues need to be considered:

1. In abstract, the research purpose is expected to be added.

It has been added. We thank the reviewer for spotting this missing point.

2. In abstract, Conclusion-“Depression was typically experienced as a reduced state of being, and was thought of cognitively as an entity that may be a part of or separate from the self. .. the results emphasized the importance of examining self-concepts in therapy and recovery.…” Such a conclusion might be relevant to young adults’ developmental task at their developmental stage? It might not be applicable for other groups at different ages?

Thank you for this. We agree with the reviewer that individuals go through different developmental stages throughout their lives and the conclusion is only relevant to young adults, which is the premise of this study. Major Depressive Disorder is the most prevalent mental illness among young adults aged 18 to 34 years old (Subramaniam et al., 2020), thus, these narratives are descriptive of how depression is typically experienced at the onset for this age group. This aspect of the abstract has been changed to elaborate this point.

3. In Introduction, please add the rationale why selecting the young adults as a target? Relevant research significance would be helpful, eg; What’s the global prevalence of major depression in young-aged papulation?

Thank you for this. Local data reports that the onset of depressive disorders lie within the age range of 18-34 years, thereby making it a relevant age range to target. By understanding how depression is experienced among young adults, it may help to inform treatment and therapy that is relevant for this age group. We agree with the reviewer that this is underdeveloped in the original draft, and have made substantial improvements. We hope the subsequent draft reads better.

4. The positive aspects of the illness perception were mentioned from several literatures (Line 48-57). Please compare your results with previous studies in Discussion.

Thank you for this. Similarly seen in past reports on anorexia nervosa and obsessive compulsive disorder, depression was not always appraised as negative (Higbed & Fox, 2010; Pedley, Bee, Wearden, & Berry, 2019)). Having depression gave new meaning and perspectives (i.e. advocacy, empathy) into the lives of some participants. We agree with the reviewer that this portion should be included in the discussion.

5. In Method, Line121-More clear information is needed, for example: interviewees’ qualification and training? Were interview process standardized or using the identical interview guidelines? What contents of interview guidelines included? Or how did you achieve the same focus of the interview contents?

Thank you for this. The interviewers and coders were trained in thematic analysis conducted by the National University of Singapore (NUS). The interview process was standardized using an interview schedule, which we had left out during the first submission; we have included the interview guide as supporting information in the subsequent draft.

6. How could the researcher prevent yourselves to be influenced or guided by these prior understanding? For example, the existing knowledge on the Common-sense Model of Self-Regulation (CSM) by Leventhal. Was the interview guideline or analytical coding process followed by the CSM theory?

Thank you for this. Before the start of qualitative inquiry, the first author and coders conducted preliminary literature review searches to attain a level of theoretical sensitivity (Mills, Bonner, & Francis, 2006). The interview guide thus follows the CSM theory.

7. Line144-150, please add the Standardized Deviation following the mean to indicate the variation and range of the sample characteristics. Most are single, please report the %.

Thank you for this. We have reported the SD and percentages in the following draft.

8. Line148, 6 participants live with depressive disorders lower than 1 year, with a mean of 3.5 years, and the least was 4 months. How will it influence your results? It might be some limitation.

Thank you for the question. We understand the reviewer’s concerns: an individual who was officially diagnosed just 4 months ago would have a different experience than another who had been officially diagnosed 16 years ago. In this study, outpatients were invited to participate regardless of the length of years diagnosed. We did not put limits on this, as deciphering the exact start points of mental illness (unlike physical illness which are more clear) is difficult unless individuals were followed through a prospective longitudinal study. Furthermore, it is not uncommon for individuals to experience a duration of untreated depression for a good number of years without their awareness, before an official diagnosis is given. Thus, this information may be obscure as well. Finally, we did not find any deviations in narratives of those who lived with depressive disorders for less than 1 year than the rest.

9. About the analysis, in Line 145-146- How was the transcript of one individual from Sri Lankan analyzed with the rest of the transcripts as a whole?

That participant, though Sri Lankan, had mentioned in the interview (and recorded in the transcript) that she had “lived in Singapore most of (her) life”. Her narrative was not substantially different from the rest, and hence, her transcript was analysed as a whole since she did not belong to any of the major ethnic groups. That is, during the meetings between coders, the themes that were coded in her transcript was analysed and reviewed together with the rest of the transcripts while ignoring her Sri Lankan ethnicity.

10. In Results, it’s not easy to distinguish the “themes” and “sub-themes”. Please clearly separate the “5-themes” and “sub-themes” to increase the readability.
11. Among all themes identified as cognitive representations, I’m wondering was there any emotional perspective or anything related to their coping or management with the mood symptoms while looking at their illness ration than cognitive aspects?

Thank you for this, questions on coping methods and management were indeed asked during the interviews (please see the interview schedule). However, we had decided to discuss this portion in a separate manuscript.

12. Line 356, “3.4.1…”?

This was an error. Thank you for spotting this.

13. Table—what’s the meaning of the 「^」 following the number, eg: 2^ or 7^?

The symbol “^” denotes self-reported approximate number of years diagnosed with depressive disorder. These participants could not remember the exact year they were diagnosed, hence, the figures reported are a rough estimation.

Thank you very much for taking the time to review this article.

--

We hope the subsequent draft reads better and we are open for further discussions and feedback. We hope to hear from you soon and thank you very much again for the time to review our manuscript.

Your sincerely,

Wen Lin

Institute of Mental Health Singapore

References cited:

Higbed, L., & Fox, J. R. (2010). Illness perceptions in anorexia nervosa: A qualitative investigation. *British Journal of Clinical Psychology, 49*(3), 307-325.

Mills, J., Bonner, A., & Francis, K. (2006). The development of constructivist grounded theory. *International journal of qualitative methods, 5*(1), 25-35.

Pedley, R., Bee, P., Wearden, A., & Berry, K. (2019). Illness perceptions in people with obsessive-compulsive disorder; A qualitative study. *PloS One, 14*(3), e0213495.

Subramaniam, M., Abdin, E., Vaingankar, J., Shafie, S., Chua, B., Sambasivam, R., . . . Chua, H. (2020). Tracking the mental health of a nation: prevalence and correlates of mental disorders in the second Singapore mental health study. *Epidemiology Psychiatric Sciences*, 1-10.
