# Supplementary material for: A reduced state of being: The role of culture in illness perceptions of young adults diagnosed with depressive disorders in Singapore
Source: PLoS One. 2021 Jun 9;16(6):e0252913. doi: 10.1371/journal.pone.0252913 (PMC8189483; doi:10.1371/journal.pone.0252913)
Supplement: Attachment — Submitted filename: plos one review-20210111.docx [file pone.0252913.s003.docx]

PONE-D-20-07514 A reduced state of being: illness perceptions in young adults diagnosed with depressive disorders

Thank you for asking me to secondly review this manuscript. Most of the comments has been well responded, but the following issues still need to be considered to make their effort valuable in cultural diversity and future implication.

1. I’m curious could it be possible that the illness perceptions and experience of your participants have not limited to the CSM framework? The author replied that their interview guide follows the CSM theory, that will easily produce an accordant result with the CSM as their prior framework. How could the researcher prevent yourselves to be mainly influenced by the prior understanding on the existing knowledge on the CSM?

2. The stigma-related issues in non-western society have been mentioned in Discussion; but it has not been reflected from any of patients’ quotations?
3. Please clarify some unclear wordings in Discussion, such as:

…that was heavily instilled “from young” at home.

…the need to further understand depression in relation to goal failure and coping cannot be better emphasized by the temporal overlap.

4. I appreciate the revision has added more quotations to reveal the cultural significance, for example: “High societal expectations of success”, and “Unable to pursue personal goals that were incongruent with familial and societal expectations”. However, would it possibly relate to the families and the society’s insufficient mental health literacy about depression? It’s suggested to refer existing evidence on the family illness perception and stigma as follows. Please add some related implication as well.

Huang CH, Li SM, & Shu BC. Exploring the relationship between illness perceptions and negative emotions in relatives of people with schizophrenia within the context of an affiliate stigma model. J. Nurs. Res 24(3), 217- 223 (2016).

Lee, S. K., Lin, E. C. L., Chang, Y. F., Shao, W. C., & Lu, R. B. (2017). Psychometric evaluation of family illness perceptions of patients with schizophrenia. *Neuropsychiatry,* *7*(7), 739-747.
