# Supplementary material for: A reduced state of being: The role of culture in illness perceptions of young adults diagnosed with depressive disorders in Singapore
Source: PLoS One. 2021 Jun 9;16(6):e0252913. doi: 10.1371/journal.pone.0252913 (PMC8189483; doi:10.1371/journal.pone.0252913)
Supplement: Attachment — Submitted filename: Letter to Reviewers 3.docx [file pone.0252913.s005.docx]

17 May 2021

Stephan Doering
Editor
PLOS ONE

Ref: PONE-D-20-07514 “A reduced state of being: the role of culture in illness perceptions of young adults diagnosed with depressive disorders in Singapore”

Dear Dr. Doering, Reviewer #3 and #4,

We appreciate the reviewers’ time and effort to review our manuscript. Please see our point-by-point responses to their comments in the boxed text-area below:

--

6. Review Comments to the Author

Reviewer #3: Thank you very much for the invitiation to review this interesting manuscript. I have noticed that both the previous review-ers as well as the authors have done a great job in improving the draft. However, I have tried to review the current version as an independent reviewer, and thus focussed my review on the manuscript rather than the previous comments.
The qualitative paper describes illness perceptions of Chinese, Malay and Indian young adults living in Singapure with depres-sive disorder. Using face-to-face semir-structured interviews, the authors concluded that participants experienced depres-sion as a reduced state of being. Further, five themes were extracted from thematic analysis. Both topic and study popula-tion seem of interest for the field, yet while I am not an expert in qualitative research and I did not review the first draft of the manuscript, I have some substantial issues with its current version - especially regarding its theoretical foundation and its overall scope.

Introduction
1.) In the introduction, the CSM is explained quite well. But the illness representations of mental illness are oversimpli-fied, and studies on bipolar disorder, schizophrenia and later anorexia nervosa combined to construct a narrative stating that “the boundary between mental illness and the self is far less clear [than in case of physical illnesses] and often intersects”. This argumentation completely neglects modern biopsychosocial approaches in medicine, foster-ing dualistic thinking. Second, egosyntonic and egodystonic concepts of different mental illnesses – including de-pression – have been discussed in clinical psychology for years.

Thank you for the comments. The biopsychosocial model explains complex individual-environment interactions in relation to illness. Research generally supports the biopsychosocial model as a dominant framework of today and its use have been widespread. Narrative or lay-person models are similar to the biopsychosocial model in that it informs the biopsychosocial aspects of illness. While the biopsychosocial model is commonly used to explain the causality of illness using a three pronged approach, the CSM is used to explain how illness is perceived by the patients themselves. In the narratives, young adults generally endorsed the biopsychosocial model of depression, however, they placed greater emphasis and salience on sociocultural elements. From a social constructivist perspective, sociocultural elements of depression may inform egosyntonic and egodystonic concepts that are relevant to a multi-cultural non-western setting, which can be highly applicable locally since existing models are predominantly western-based. Qualitative approaches thus allow for the voice of culture to be heard. We respectfully disagree with the reviewer indicating that the CSM neglects the biopsychosocial model completely. Instead, both models do overlap, and we believe that the current focus on narratives can inform, substantially, in areas that the biopsychosocial model overlooks, such as culture.

2.) The overview of the existing qualitative literature on the topic also seems somewhat random. There are numerous qualitative studies on depression, for example, while the manuscript suggests that “the majority of qualitative work has been conducted amongst severe but less common forms of mental illness such as psychosis or schizophrenia”. This might be true with regard to illness representations, it is however important to be more precise with the re-search question you want to investigate in the manuscript, and what previous research you built your argumenta-tion upon.

While it may be true that there are numerous qualitative studies that had explored depression in its broadest form, qualitative inquiry into illness narratives of depression in young adults in South-east Asia are few and far between. Most of existing research on illness perception (cited) have been documented in western communities, and the extent to which it is relatable to a multi-ethnic non-western setting may be questioned. While we did expect to find illness narratives to be highly associated with the self and self-concepts as reported by past research, our research questions (and of past research) remained broad rather than specific to capture cultural nuances. Prompts were made to improve the specificity of our interview schedules but they may depend very much on the direction and content of narratives.

3.) When I was reading the sentence “To the best of our knowledge, there has been no qualitative inquiry into illness perceptions of depression that is specific to a non-western psychiatric population residing in Singapore”, I felt like this is the main novelty the manuscript provides. However, reading the title as well as the abstract, I got a com-pletely different first impression of the manuscript’s aims and hypotheses. I think you should really focus on the main research question, rather than generalize qualitative results from a very specific population. For example, I found the description of the multi-ethnic Singapore population really interesting and as an important context factor for the interviews.

We agree with the reviewer that the existing title does not encapsulate the intention and results of our study. We have made further changes to the title to capture our work better.

4.) I do not really understand from the introduction why you focused on young adults, apart from them being the “most pervasive” age group. I think a group of young people living in Singapor suffering from depression is a very in-teresting, yet also very specific group of patients. I think your work would benefit a lot from focusing on the particu-lar cultural and social insights you may get from this group, rather than trying to generalize the results on depres-sion (or even mental illness) as an entity.

Young adulthood is arguably the period where an individual is most productive in life and from an economic standpoint, mental illness can pose a significant threat to productivity and disease burden. This is crucial for Singapore as human capital is considered an important asset. Thus, we have focused on young adults due to this overlap. We have since elaborated our study’s rationale in the next draft.

We have added 3 new references to support the claims regarding the importance of culture in treatment relevance for young adulthood, citation [31]: McGorry PD, Goldstone SD, Parker AG, Rickwood DJ, Hickie IB. Cultures for mental health care of young people: an Australian blueprint for reform. The Lancet Psychiatry. 2014;1(7):559-68;

Human capital in Singapore, citation [32] Cheng YE. Cultural Politics of Education and Human Capital Formation: Learning to Labor in Singapore. In: Abebe T, Waters J, Skelton T, editors. Laboring and Learning. Singapore: Springer Singapore; 2017. p. 265-84; and

Treatment gaps in Singapore pertaining to mental illness: citation [34] Subramaniam, M., Abdin, E., Vaingankar, J. A., Shafie, S., Chua, H. C., Tan, W. M., ... & Chong, S. A. (2019). Minding the treatment gap: results of the Singapore Mental Health Study. Social psychiatry and psychiatric epidemiology, 1-10.

5.) The first reseach question if far too general, and there are numerous (clinical) works on the illness perceptions in (young) persons with depression. As you are not able to answer it with your data, I would at least add “in Singapore” to it, and then elaborate on specific cultural influences in the second research question.

We have included the terms “Singapore”, and “locally” to be more specific, we thank the reviewer for the suggestions.

6.) I found the last paragraph in the introduction (your hypotheses?) to be very confusing, as you only give general statements rather than linking them to either your research questions or your data.

Thank you for spotting this, while we do expect to find similar themes that were reported in past literature, we refrained from the terms hypothesis testing as the nature of the study is exploratory.

Methods
7.) Please state what “IMH” stand for.

Thank you for spotting this. The IMH stands for the Institute of Mental Health, the only public state psychiatric hospital in Singapore.

8.) I am rather confued by the interviews taking place “at a convient place and time”, but then again in the research in-terview room in IMH “to minimize the possibility that participants may describe their illness beliefs in medical terms”. Also, you matched interviewers and patients by ethnicity, without providing any rational why. Further, in-clusion criteria included ability to read and speak English, yet interviews were only “primarily” conducted in English. While there might be many good and interesting reasons to conduct the qualitative interviews the way you did, from a scientific point of view it all sounds very arbitrary.

Thank you for noting the contradictions. By default, researchers conducted the interviews in the research rooms that were situated away from the clinic. However, if a participant is unwilling, we would interview the participant at a time and place that is convenient for the him/her. The interviews were conducted in English, but participants, as with all young adults in Singapore, are bilingual, therefore, code-switching to Mandarin, Malay, Tamil, or other languages may happen during the interview. This brings us to our final point on cultural sensitivity. Interviewees were matched with interviewers as much as possible to minimize potential barriers from the lack of cultural sensitivity that may present itself during the interview. We have made changes to the method section to clear this contradiction and we hope it reads better in the next draft.

9.) I think you should provide some information on the interviewers as well (medical doctors, researchers, psycholo-gists?).

The authors (WLT, ES, LC, RK, FD, SS) are all quantitative researchers formal training (degrees in psychology/sociology at least), and we have had since received professional training and are experienced in the use of thematic analysis. Thank you for this suggestion. We have state this in the next draft.

10.) Were there any other (methodological) reasons for using thematic analysis by Clarke and Braun rather than the au-thors being familiar with it?

Yes, we chose this method of analysis because of the advantages it has for researchers working in healthcare. Thematic analysis has several advantages that is favourable for us in overcoming methodological constraints, such as a lack of time, resources, and expertise that are required for many other qualitative approaches. We have included these reasons in the next draft.

Results
11.) I think this section would highly benefit from a figure or an additional numbering, though this is mainly a visual re-mark.

We have included additional numberings and we hope it helps to read better in the next draft.

12.) At no point you mentioned the fact that only two of your participants were married. Did this emerge as a topic throughout the interviews? If not, this should be addressed oin the discussion?

Thank you for this comment. In Singapore, the median age at the time of first marriage is around 30 years old for men and 29 years old for women. As the average age of our participants is approximately 26 years old, it was expected that being married would not be a common attribute. Further, we did not find emergent or significant themes related specifically to being married in this study as only a few of whom were married. As only the prominent themes were discussed, we had not reported them.

Discussion
13.) In accordance to 12, you should elaborate further on the specifics of your study population.

The specifics of our study population are elaborated in the “Participants” subsection under “Methods”.

14.) I found it interesting to read that depression is “a chronic condition with no means of full recovery”; was this a gen-eral statement or a point of view maintained by all interviewees?

It was both. It was a general statement and point of view maintained by the majority of our interviewees regardless of ethnicity. They had initially hoped depression could be resolved in an finite period of time, but they had soon felt it was not the case.

15.) I enjoyed the explanations on the peculiarities of Singaporian culture. As you have a very special study population, I would be careful regarding over-generalizing your findings, though – especially regarding clinical implications and therapy. Accordingly, I would elaborate further on the weaknesses and limitations of the study .

Generalization is not the aim of qualitative research. As reviewer #1 had pointed out, “*the aim of qualitative research is not generalization but on the contrary, considering variablity, contextualization, narratives, singularity, experience - therefore it is not a limitation from my view*”, which we had agreed with.

Thank you for taking the time to review our manuscript.

Reviewer #4: PONE-D-20-07514R2. A reduced state of being: illness perceptions in young adults diagnosed with depressive disorders

This is a very interesting paper handling illness perceptions in young adults in a relatively understudied populations, which makes that the paper contributes to the literature. The paper has been reviewed before and the reviewers provide valuable suggestions which have been answered appropriately by the authors. As a newly, more recent added reviewer I will not go into the points raised before. I really appreciated the wide range of quotes of the participants given in the result section to support the categories reported on.

Minor points:
The paper’s impact on cultural significance is not reflected in the title of the manuscript. I suggest to revise the title to make the paper easier findable for potential interested readers.

Thank you for this suggestion. We have made slight changes to better encapsulate the significance of culture in this manuscript.

Abstract: …surviving with depressive disorders… sounds unfamiliar to me. Do you mean ..remitted .. here?

Yes, we do mean remitted and have changed that term.

Ending the discussion with a paragraph on the limitations of the study is not elegant and makes it more difficult for the reader to find the take home message of the paper. Please end the discussion with a concluding paragraph reflecting on the research questions given in the introduction of the manuscript (so without including speculations that cannot be derived from the current results).

We have added a paragraph to properly conclude and summarise the study’s aims and findings. Thank you for taking the time to review our manuscript.

--

Thank you.

Sincerely,

Wen Lin
